# Preventing cervical cancer using HPV self-sampling: direct mailing of test-kits increases screening participation more than timely opt-in procedures - a randomized controlled trial

**DOI:** 10.1186/s12885-018-4165-4

**Published:** 2018-03-09

**Authors:** Mette Tranberg, Bodil Hammer Bech, Jan Blaakær, Jørgen Skov Jensen, Hans Svanholm, Berit Andersen

**Affiliations:** 1 Department of Public Health Programmes, Randers Regional Hospital, Skovlyvej 15, 8930 Randers, NØ Denmark; 2 Department of Clinical Medicine, Aarhus University, Palle Juul-Jensens Boulevard 82, 8200 Aarhus N, Denmark; 3 Department of Public Health, Section for Epidemiology, Aarhus University, Bartholins Allé 2, 8000 Aarhus C, Denmark; 4 Department of Obstetrics and Gynecology, Odense University Hospital, Sdr. Boulevard 29, 5000 Odense C, Denmark; 5 Department of Clinical Medicine, University of Southern Denmark, J.B. Winslows Vej 25, 5000 Odense C, Denmark; 6 Statens Serum Institut, 2300 Copenhagen S, Artillerivej 5 Denmark; 7 Department of Pathology, Randers Regional Hospital, Østervangsvej 48, 8930 Randers, NØ Denmark

**Keywords:** Self-sampling, Cervical cancer screening, Cancer prevention, Screening participation, Human papillomavirus testing

## Abstract

**Background:**

Cervical cancer screening participation remains insufficient in most countries. Our aim was to evaluate whether offering a HPV self-sampling kit, either mailed directly to the woman’s home or using timely opt-in procedures for ordering the kit, increased screening participation compared with a standard second reminder.

**Methods:**

In this randomized, controlled effectiveness trial, 9791 Danish women aged 30–64 who were due to receive the second reminder were equally randomized to either: 1) direct mailing of a second reminder and a self-sampling kit (directly mailed group); 2) mailing of a second reminder that offered a self-sampling kit to be ordered by e-mail, text message, phone, or webpage (opt-in group); or 3) mailing of a second reminder to attend regular cytology screening (control group). In an intention-to-treat analysis, we estimated the participation rate at 180 days post intervention, by returning a self-sample or attending regular cytology screening. We calculated the proportion of women with a positive HPV self-sample who attended for cervical cytology triage at the general practitioner within 90 days.

**Results:**

Participation was significantly higher in the directly mailed group (38.0%) and in the opt-in group (30.9%) than in the control group (25.2%) (participation difference (PD): 12.8%, 95% CI: 10.6–15.0% and PD: 5.7%, 95% CI: 3.5–7.9%, respectively). Within 90 days, 107 women (90.7%, 95% CI: 83.9–95.3%) with a HPV-positive self-sample attended follow-up.

**Conclusions:**

Offering the opportunity of HPV self-sampling as an alternative to regular cytology screening increased participation; the direct mailing strategy was the most effective invitation strategy. A high compliance with follow-up was seen.

**Trial registration:**

Current Controlled Trials NCT02680262. Registered 10 February 2016.

## Background

Organized screening programs have contributed to a decline in cervical cancer incidence and mortality in many Western countries [1–3]. The magnitude of the preventive effect of cervical cancer screening depends on high participation and coverage, as well as timely follow-up and treatment of premalignant lesions [4, 5]. However, more than half of all invasive cervical cancers diagnosed in countries with organized screening programs arise among under- or unscreened women [6–8]; thus, targeting non-participating women is crucial.

Common barriers to cervical cancer screening include discomfort during the pelvic examination and inconvenient appointment times [9, 10]. The on-going introduction of high-risk human papillomavirus (hrHPV) testing in cervical cancer screening [11, 12] now makes it possible to overcome these barriers by offering women a test-kit for home-based cervico-vaginal self-sampling for hrHPV testing (HPV self-sampling). Self-samples have shown a sensitivity in detecting cervical intraepithelial neoplasia grade 2+ (CIN2+) that is similar to that of clinician-collected samples, provided that certain validated PCR based HPV DNA tests are used [13, 14]. Generally, self-sampling enjoys acceptance among women [15].

Mailing self-sampling kits directly to women’s home addresses has been shown to improve participation in cervical cancer screening compared to the regular screening invitation/reminder [16], with participation rates ranging from 10% to 39% among underscreened women [17, 18]. To minimize the number of wasted kits and associated costs, other trials [19–21] have explored the effect of offering opt-in self-sampling, i.e. receiving an invitation to actively order the kit by phone [20], by ordinary mail [19], or by picking it up at the pharmacy [21]. The results of these trials are mixed; one trial reported a 13.9% increase in participation among long-term non-participants [19], while two other trials found no positive effect [20, 21]. The opt-in strategy may be more effective if the kit can be ordered electronically via e-mail request, webpage, or text message (i.e. timely opt-in procedures) owing to greater convenience. However, one cluster-randomized trial showed no effect on participation among young women, although the kit could be requested by e-mail and text message [22]. Thus, more trials are warranted to explore the effectiveness of timely opt-in procedures and direct mailing of the kit before implementing the optimal self-sampling invitation strategy to increase screening participation in organized programs. Furthermore, a HPV-positive self-sampling result should be accompanied by appropriate follow-up to make the screening offer beneficial, but compliance to follow-up after a HPV-positive self-sampling result has varied widely (range 41 to 100%) [19, 23].

Within the context of a routinely organized screening program, our aim was to compare the effect on screening participation of mailing a self-sampling kit directly to women and timely opt-in procedures for ordering the kit as compared with the standard second reminder for regular cytology screening. We also estimated the proportion of women with a HPV-positive self-sample undergoing the recommended follow-up testing.

## Methods

The present study was conducted in line with the protocol of the CHOiCE (Cervical HOme-based CancEr screening) trial published elsewhere [24]. The report of this clinical trial conforms to the CONSORT statement [25].

### Design and study setting

CHOiCE was a randomized, controlled, effectiveness population-based trial, nested in the Danish organized cervical cancer screening program conducted in the Central Denmark Region between March 2016 and May 2017 [24]. This region is a mixed rural and urban area, covering approximately one-fourth of the Danish population [26].

In Denmark, cervical cancer screening is organized as a nationwide integrated program. The program is based on a call-recall system using data from the invitation module in the Danish Pathology Data Bank (DPDB) [27]. This module keeps track of women who are due to receive invitations and reminders to participate in screening, and it contains information on women who are no longer subscribed to the screening program, e.g. due to hysterectomy. Routinely, all pathology specimens including cervical cytology results, HPV test results, and histological diagnoses from cervical biopsies are recorded in the DPDB using women’s unique civil personal registration (CPR) number [28, 29].

Danish women are recommended to participate in the screening program every third year when aged 23–49 years and every fifth year when aged 50–64 years [30], but opportunistic testing is frequent [31]. Based on their screening status in the DPDB, only women not registered with a cervical cytology sample within the recommended time interval are invited for screening. The invitation is sent to the woman’s home address advising her to book an appointment to have a liquid-based cervical cytology specimen collected by her general practitioner (GP) [30]. Hereafter, liquid-based cervical cytology is referred to as cervical cytology.

The cervical cytology specimen is mailed to the local department of pathology for analysis. If no cervical cytology is registered in the DPDB, up to two reminders will be sent at 3 and 6 months after the initial invitation [30]. If no cervical cytology is taken within 3 or 5 years after the initial invitation, the woman is sent a new invitation in the next screening round, unless she has actively opted out of the screening program. In Denmark, screening and treatment are provided free of charge.

In the Central Denmark Region, the Department of Pathology, Randers Regional Hospital analyzes all samples obtained in connection with the Cervical Cancer Screening Program. Invitations and reminders are routinely handled by the Department of Public Health Programs, Randers Regional Hospital [24].

### Study population and randomization

Included in the study were women aged 30 to 64 years who were due to receive the second reminder from the Central Denmark Region between March 7th 2016 and August 8th 2016. All eligible women were identified on a weekly basis in the invitation module in the DPDB, and no exclusion criteria were used.

Web-based computer randomization in RedCap was used to allocate eligible women to the three groups of the trial at a 1:1:1 ratio by the method of individual randomization with randomly varying block sizes of 3, 6, and 9 [32]. The randomization list was produced by an independent programmer who was not otherwise involved in the trial. The women were unaware of the randomization, but blinding of the participants and study staff was impossible due to the nature of the interventions.

### Interventions

Women in the directly mailed group received a modified second reminder, a leaflet, and a self-sampling kit. The modified second reminder informed of the possibility of collecting a self-sample, but also about the possibility of having a cervical cytology specimen taken at the GP [24]. The kit included a brush device (Evalyn® Brush, Rovers Medical Devices B.V, Oss, Netherlands) to collect a cervico-vaginal sample for subsequent hrHPV testing [33]. The kit also included instructions describing how to obtain and mail the sample as well as a pre-stamped return envelope addressed to the Department of Pathology, Randers Regional Hospital, which performed the hrHPV testing [24].

Except for the kit, women in the opt-in group received the same material as those in the directly mailed group [24]. Additionally, the leaflet for this group held information describing how to order the kit by e-mail, text message, phone, or via a study webpage (www.hjemme-us.rm.dk) [24]. After receiving the orders in our department, the self-sampling kit was mailed to the women within 2 to 4 working days.

**Fig. 1:**
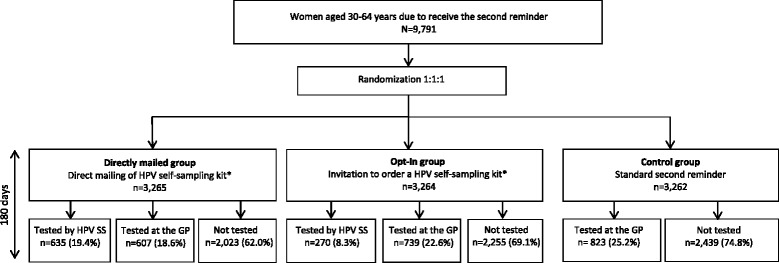
Flow chart of the study design. Abbreviations: HPV self-sampling: HPV SS. GP: General Practitioner.*) Also had the possibility of attending a GP for regular cytology screening

Women in the control group received a standard second reminder that informed them about the current test opportunity [24]. The reminder contained no information about self-sampling [24]. All study material was written in Danish. Throughout the study, a telephone helpline and a webpage with information for the GPs were available. Figure 1 shows the study design.

### Analyses of samples

Upon arrival at the Department of Pathology, the dry brush head was placed in 10 ml of SurePath medium (BD Diagnostics, Burlington, NC), stored overnight, and then vortexed for 5 min. Following this, 2 ml of the medium was placed in a test tube, which was the starting point for hrHPV testing. The brush specimens were tested for hrHPV using the clinically validated and Federal Drug Agency (FDA)-approved Cobas® 4800 HPV DNA test (Roche Diagnostics, GmBH, Switzerland), according to the manufacturer’s instructions. This test is designed to detect HPV16, HPV18, and 12 other hrHPV types (31, 33, 35, 39, 45, 51, 52, 56, 58, 59, 66 and 68) in a single pool [34]. Results were either 1) hrHPV negative, 2) hrHPV positive (HPV16, HPV18 and/or other hrHPV types), or 3) invalid [24]. The hrHPV test results of the self-samples were registered in the DPDB [24].

Cervical cytology specimens obtained by GPs were taken with a cervical brush, stored in 10 ml SurePath medium (BD Diagnostics, Burlington, NC), and mailed to the Department of Pathology. The specimens were analyzed using the standard procedure in the Central Denmark Region [30], i.e. relying on cervical cytology using the Bethesda nomenclature for control group women aged 30–59 years. Women with ASC-US have reflex Cobas® 4800 (Roche Diagnostics, GmBH, Switzerland) hrHPV triage. For women aged 60–64 years, the primary analysis was hrHPV testing using the Cobas® 4800 (Roche Diagnostics, GmBH, Switzerland) test, followed by cytology to triage women positive for other hrHPV types than HPV16/18 for onward referral. Women positive for HPV16/18 were referred directly to colposcopy, whereas hrHPV-negative women left the screening program.

### Follow-up algorithm after self-sampling

Test results and follow-up recommendations for the self-samples were mailed to the women and their GPs, unless the woman had rejected that option.

Women with a hrHPV-positive self-sample test result were advised to visit their GP for a cervical cytology triage specimen within 30 days [24]; after this, the GP should handle the women as described in Danish routine guidelines, i.e. women with abnormal cervical cytologies (threshold ASC-US and hrHPV positive or LSIL, or ASC-H or HSIL or AGC, or AIS or malign tumor cells) should be referred to a gynecologist for colposcopy within 3 months, women with normal cervical cytologies were invited for a cervical cytology sample and hrHPV retesting after 12 months, and women with inadequate cervical cytologies were recommended to have a new cytology sample taken within 2 to 4 months [30]. At colposcopy in a gynecological clinic, cervical lesions should be biopsied and/or treated according to routine Danish guidelines and sent to the Department of Pathology, which classifies the samples using the CIN nomenclature [30].

In cases with a hrHPV-negative self-sampling test result, the women were referred back to the national screening program with a recommendation to participate in the next screening round. Women with an invalid self-sample received a second self-sampling kit and were encouraged to repeat self-sampling at home or to visit a GP for regular cytology screening [24].

### Outcomes

The primary outcome was the participation rate, i.e. the percentage of randomized women who returned a self-sample or attended regular cytology screening at their GP within 180 days (i.e. 6 months) post second reminder [24]. Secondary outcomes included the prevalence of hrHPV among self-samplers and the percentage of women with an hrHPV-positive self-sample having a recommended follow-up. Follow-up was defined as attending a GP for a cervical cytology triage sample within 30, 60, or 90 days [24]. Further outcomes measured were use of the different opt-in procedures and the proportion of histologically confirmed CIN2+ lesions (including CIN2, CIN3/AIS and carcinoma) in the three groups. Categorization of the histological diagnosis was based on the most severe diagnosis if further histological diagnosis was available.

### Sample size

The sample size was determined based on the ability to detect differences of 3.6% in the participation rate between the intervention groups and the control group [35]. To achieve a statistical power of 80% while assuming that the participation rate in the control group was 28.7% [36], we had to include at least 3109 women in each group (a total of 9327 women) [24]. Kits and reminders were sent out progressively on a weekly basis, and this process was to continue until at least 9327 women had been included in the study [24].

### Classification of screening history

Each woman’s screening history over the previous 15 years was determined through linkage to the DPDB using data from January 1th 2001 to March 7th 2016. All registered cervical cytology samples in the period were included in the analysis. We calculated if a woman had participated in the previous screening round and we registered the number of cervical cytology samples during the previous 15 years. Women aged 29 to 49 and 50 to 64 years at the time of initial invitation (i.e. 6 months prior to the age at the time of the second reminder) were defined as having been screened in the previous screening round if a cervical cytology result had been registered within the past 3.5 years and 5.5 years, respectively.

Women aged 30 to 34 years (7 to 11 years of screening history) or 56 to 64 years (15 years of screening history) were defined as “regularly screened” if ≥2 cervical cytology samples were registered, and as “underscreened” if they had only one cervical cytology sample registered. Women aged 35 to 55 years (12 to 15 years of screening history) were defined as “regularly screened” if ≥3 cervical cytology samples were registered, and as “underscreened” if they had only one or two cervical cytology sample(s) registered. Women in all ages were defined as being “unscreened” if no cervical cytology sample was registered.

### Statistical analysis

Descriptive statistics (numbers and proportions) were used to compare the baseline characteristics of the women in the intervention groups and the control group. The participation rate in each group was calculated using intention-to-treat analysis. Participation rates in the directly mailed group and the opt-in group were compared to those of the control group by estimating both the absolute difference in participation rate (PD) and the ratio followed by 95% confidence intervals (CIs). The analyses were also stratified by age and screening history. We estimated the hrHPV prevalence among self-samplers and the number of histologically confirmed CIN2+ lesions in the groups. Estimates and 95% CIs for the proportion of women attending the recommended follow-up were calculated.

In a sensitivity analysis, we assessed the impact on the participation rates of giving women a shorter time frame to respond to the interventions. Thus, we used 90 days (instead of 180 days) post second reminder as the cut-off value to differentiate between participants and non-participants. This approach is in accordance with the definition used to determine the participation rate after the second reminder in the Danish Quality Database for cervical cancer screening [37].

All analyses were performed using STATA version 13 (College Station, TX: StataCorp LP).

### Ethical statement

The study was approved by the Danish Data Protection Agency (j.no: 1–16–02-495-15) and by the Danish Health Authorities (j.no: 3–3013-1407/1). Furthermore, the study achieved clearance at the Central Denmark Region Committees on Health Research Ethics (j.no: 1–10–72-259-15). The included women were informed that if they returned their self-sample they thereby expressed their consent to the analysis of the sample and to receiving any test results and follow-up recommendations by mail.

## Results

### Study population

A total of 9791 women were included in the study and randomized into three groups: 3265(33.3%) in the directly mailed group, 3264 (33.3%) in the opt-in group, and 3262 (33.3%) in the control group (Fig. 1). The distribution of age and screening history was similar across the three groups (Table 1).

**Table 1:** Baseline characteristics of the study population

	Directly mailed group *N* = 3265	Opt-in group *N* = 3264	Control group *N* = 3262
	n (%)	n (%)	n (%)
Age (years)			
30–39	1236 (37.9)	1215 (37.2)	1251 (38.4)
40–49	1300 (39.8)	1353 (41.5)	1342 (41.1)
50–64	729 (22.3)	696 (21.3)	669 (20.5)
Screened in the previous screening round^a^			
Not screened	1298 (39.8)	1272 (38.9)	1249 (38.3)
Screened	1967 (60.3)	1992 (61.0)	2013 (61.7)
Screening history^a^			
Unscreened	604 (18.5)	595 (18.2)	567 (17.4)
Underscreened	802 (24.6)	842 (25.8)	842 (25.8)
Regularly screened	1859 (56.9)	1827 (56.0)	1853 (56.8)

n: Number of randomized women. %: Column percentages. ^a^See classification of screening history for definitions

### Participation

A total of 1242 (38.0%) women from the directly mailed group participated after the second reminder, 635 (19.4%) by returning the self-sample, while the remaining 607 (18.6%) attended regular cytology screening (Table 2). This was 12.8% (95% CI: 10.6–15.0%) more than the 25.2% participating in the control group. The opt-in strategy resulted in a total participation of 1009 women (30.9%): 270 (8.3%) participated by returning a self-sample, and 739 (22.6%) attended regular cytology screening. Thus, the total participation in the opt-in group was lower than in the directly mailed group; still it was significantly higher than in the control group (PD: 5.7%, 95% CI: 3.5–7.9%) (Table 2). In all three groups, the participation rate was lowest for women aged 50–64 years.

**Table 2:** Participation rate, stratified by age groups and screening history

		Directly mailed group						Opt-in group					Control group	
		Self-sampling	Cytology	Total^a^				Self-sampling	Cytology	Total^a^				Cytology
	N	n (%)	n (%)	n (%)	PD^1^ (95% CI)	RR^1^ (95% CI)	N	n (%)	n (%)	n (%)	PD^2^ (95% CI)	RR^2^ (95% CI)	N	n (%)
Age (years)														
30–39	1236	231 (18.7)	264 (21.4)	495 (40.0)	13.4 (9.8–17.1)	1.50 (1.34–1.69)	1215	81 (6.7)	316 (26.0)	397 (32.7)	6.1 (2.5–9.7)	1.23 (1.09–1.39)	1251	333 (26.6)
40–49	1300	271 (20.9)	260 (20.0)	531 (40.8)	13.3 (9.8–16.9)	1.49 (1.33–1.66)	1353	126 (9.3)	326 (24.1)	452 (33.4)	5.9 (2.4–9.4)	1.21 (1.08–1.36)	1342	369 (27.5)
50–64	729	133 (18.2)	83 (11.4)	216 (29.6)	11.5 (7.1–15.9)	1.63 (1.35–1.99)	696	63 (9.1)	97 (13.9)	160 (22.9)	4.9 (0.6–9.2)	1.27 (1.03–1.57)	669	121 (18.1)
Total	3265	635 (19.4)	607 (18.6)	1242 (38.0)	12.8 (10.6–15.0)	1.51 (1.40–1.62)	3264	270 (8.3)	739 (22.6)	1009 (30.9)	5.7 (3.5–7.9)	1.23 (1.13–1.32)	3262	823 (25.2)
Screened in previous screening round^b^														
Not screened	1298	203 (15.6)	97 (7.5)	300 (23.1)	14.9 (12.2–17.7)	2.83 (2.29–3.49)	1272	76 (5.9)	95 (7.5)	171 (13.4)	5.2 (2.9–7.7)	1.65 (1.30–2.08)	1249	102 (8.2)
Screened	1967	432 (21.9)	510 (25.9)	942 (47.9)	12.1 (9.0–15.1)	1.34 (1.24–1.44)	1992	194 (9.7)	644 (32.3)	838 (42.1)	6.3 (3.2–9.3)	1.17 (1.09–1.27)	2013	721 (35.8)
Screening history^b^														
Unscreened	604	81 (13.4)	39 (6.5)	120 (19.9)	12.6 (8.8–16.4)	2.75 (1.96–3.84)	595	16 (2.7)	36 (6.1)	52 (8.7)	1.5 (−1.6–4.6)	1.21 (0.82–1.79)	567	41 (7.2)
Underscreened	802	135 (16.8)	78 (9.7)	213 (26.6)	12.3 (8.4–16.2)	1.86 (1.52–2.28)	842	71 (8.4)	97 (11.5)	168 (19.9)	5.7 (2.1–9.3)	1.4 (1.13–1.73)	842	120 (14.3)
Regularly screened	1859	419 (22.5)	490 (26.4)	909 (48.9)	13.2 (10.0–16.3)	1.37 (1.27–1.48)	1827	183 (10.0)	606 (33.2)	789 (43.2)	7.5 (4.3–10.6)	1.21 (1.12–1.31)	1853	662 (35.7)

Participation rate is calculated as having a self-sample or a cervical cytology sample within 180 days after the second reminder. N: Number of randomized women. n: Number of participants in the group

%: Row percentages. ^a^Total number of returned self-samples and cervical cytology samples. ^b^See classification of screening history for definitions

Abbreviations:

PD^1^: Participation difference in the total participation in the directly mailed group compared to the total participation in the control group

RR^1^: Total participation in the directly mailed group compared to the total participation in the control group

PD^2^: Participation difference in the total participation in the opt-in group compared to the total participation in the control group

RR^2^: Total participation in the opt-in group compared to the total participation in the control group

### Participation in relation to screening history

The offer of self-sampling was effective in motivating both un- and underscreened women to participate (Table 2). A higher participation was seen in the directly mailed group than in the control group among women who had not participated in the previous screening round (PD: 14.9%, 95% CI: 12.2–17.7%). A similar, though less pronounced, tendency was seen in the opt-in group (PD: 5.2%, 95% CI: 2.9–7.7%).

Among un- and underscreened women, participation was significantly higher in the directly mailed group than in the control group (PD: 12.6 and 12.3%, respectively). In the opt-in group, we found no significant effect among unscreened women (PD: 1.5%, 95% CI: -1.6-4.6%); a significant difference in the participation rate was found only for underscreened women (PD: 5.7%, 95% CI: 2.1–9.3%).

### Effect on the overall participation rate in the screening program

The overall participation rate after the initial invitation and the first reminder was estimated to be 58% among the targeted women: thus, the overall participation after the invitation and two reminders would reach 74.0% (95% CI: 73.6–74.4%) in the directly mailed group, 71.0% (95% CI: 70.6–71.4%) in the opt-in group, and 69.0% (95% CI: 68.6–69.4%) in the control group. By offering the possibility of self-sampling to non-participants, the overall participation rate among invited 30–64 year-old women would increase by 5.0% (95% CI: 4.8–5.2%) (from 69.0% to 74.0%) if the direct mailing strategy was used and by 2.0% (95% CI: 1.9–2.1%) if the opt-in strategy was used (from 69.0% to 71.0%) (data not shown).

### Ordering of the self-sampling kit in the opt-in group

In the opt-in group, the self-sampling kit was ordered by a total of 409 women of whom 270 (66.0%) returned the sample within 180 days. Seven women (1.7%) ordered the kit but attended a GP for regular cytology screening, while 132 women (32.3%) ordered the kit but were not tested within 180 days. The majority of women ordered the kit through the webpage (70.0%) or by sending a text message (19.8%). Phone and e-mail were rarely used (7.8% and 2.4%, respectively) (data not shown).

### HPV prevalence and compliance with follow-up among self-samplers

Of the 905 self-samples (including samples from both intervention groups), three samples (0.3%) were invalid for hrHPV testing, 118 (13.0%, 95% CI: 10.9–15.4%) were hrHPV positive, and the remaining 784 samples (86.6%) were hrHPV negative (Table 3). Most women were positive for other hrHPV types than HPV16/18 (65.2%), followed by HPV16 (12.7%), and HPV18 (9.3%) (data not shown). The proportion of hrHPV-positive women decreased with age from 15.7% at age 30–39 to 10.7% at age 50–64 (Table 3).

**Table 3:** HrHPV prevalence and compliance with follow-up in self-samplers, stratified by age groups

	HrHPV prevalence		Compliance with follow-up within (days)			Total
			≤ 30	31–60	61–90	
	N	n^a^ (%)	n^b^(%)	n^b^(%)	n^b^(%)	n^b^(%)
Age (years)						
30–39	312	49 (15.7)	40 (81.6)	2 (4.1)	2 (4.1)	44 (89.8)
40–49	397	48 (12.1)	31 (64.6)	9 (18.8)	4 (8.3)	44 (91.7)
50–64	196	21 (10.7)	11 (52.4)	7 (33.3)	1 (4.8)	19 (90.5)
Total	905	118 (13.0)	82 (69.5)	18 (15.3)	7 (5.9)	107 (90.7)

N: Number of received self-samples. n^a^: Number of women with a hrHPV-positive self-sample. n^b^: Number of women with a cervical cytology triage sample

%: Row percentages

Compliance with the follow-up cervical cytology triage sample at the GP within 90 days after a hrHPV-positive result was 90.7%, (95% CI: 83.9–95.3%). Notably, more than half (69.5%) of the women attended follow-up within 30 days after sending the test results and were therefore compliant with the recommendation (Table 3). Six women (5.1%) attended follow-up 91–180 days after receiving the HPV test results, corresponding to an overall “long-term” compliance rate of 95.8%, (95% CI. 90.4–98.6%). Five women (4.2%) without follow-up had not been screened in the previous screening round and three were unscreened (data not shown). Most of the non-compliant women were from the directly mailed group (*n* = 4).

### Detection of CIN2+

The proportion of CIN2+ lesions per 1000 invited women was 5.8 (95% CI: 3.5–9.1) in the directly mailed group and 4.0 (95% CI: 2.1–6.8) in the opt-in group, compared with 3.1 (95% CI: 1.5–5.6) in the control group (Table 4). Most of the CIN2+ lesions were found in women aged < 50 years (data not shown).

**Table 4:** Yield of CIN2+ lesions in the intervention and control groups

	Directly mailed group Invited = 3265			Opt-in group Invited = 3264			Control group Invited = 3262
	Self-sampling	Cytology	Total	Self-sampling	Cytology	Total	Cytology
	n	n	n	n	n	n	n
Number screened	635	607	1242	270	739	1009	823
CIN2+ detected	13	6	19	5	8	13	10^a^
CIN2+ per 1000 invited (95% CI)	4.0 (2.1–6.8)	1.8 (0.7–4.0)	5.8 (3.5–9.1)	1.5 (0.5–3.6)	2.5 (1.1–4.8)	4.0 (2.1–6.8)	3.1 (1.5–5.6)
CIN2+ per 1000 screened (95% CI)	20.5 (10.9–34.8)	9.9 (3.6–21.3)	15.3 (9.2–23.8)	18.5 (6.0–42.7)	10.8 (4.7–21.2)	12.9 (6.9–21.9)	12.2 (5.8–22.2)

CIN2+: CIN2, CIN3/AIS and carcinoma. ^a^One case of squamous cell carcinoma

For self-samplers, the proportion of CIN2+ lesions was 20.5 (95% CI: 10.9–34.8) in the directly mailed group and 18.5 (95% CI: 6.0–42.7) in the opt-in group per 1000 women screened by self-sampling. In the control group, the proportion of CIN2+ lesions was 12.2 (95% CI: 5.8–22.2) per 1000 women screened by attending regular cytology screening (Table 4).

### Sensitivity analysis

When we used a cut-off value of ≤90 days (instead of 180 days) to differentiate participants from non-participants, the intervention groups maintained a significantly higher participation rate than the control group (directly mailed: PD: 13.4%, 95% CI: 11.4–15.5%, and opt in: PD: 4.9%, 95% CI: 3.0–6.9%) (data not shown).

## Discussion

### Main findings

In this randomized, controlled effectiveness trial, we found that directly mailing a HPV self-sampling kit to women not participating in cervical cancer screening after an invitation and one reminder resulted in significantly higher participation (38.0%) than using a timely opt-in strategy (30.9%) or a standard second reminder (25.2%) to approach their GP for regular cytology screening. Among un- or underscreened women, the direct mailing of a self-sampling kit proved to be more effective than both the opt-in strategy and the standard second reminder. Compliance to follow-up within 90 days among self-samplers was high (90.7%).

### Strengths and limitations

A major strength of this study is the randomized design that contributes to making the detected differences between the tested interventions and usual care trustworthy. Another key strength is the effectiveness approach and the fact that the study was embedded in an ongoing routine cervical cancer screening program, which from an implementation point of view provides a reliable and representative estimate of the expected participation rates that could be obtained if the possibility of self-sampling together with the second reminder were to become an option. Furthermore, we minimized the risk of information bias and selection problems, firstly, by using data from the DPDB which has highly valid records on all pathology specimens for identifying outcomes and, secondly, by using a population-based design. Additionally, we used a combination of a clinically validated self-sample device and a clinically validated PCR-based HPV DNA test, resulting in a low proportion of invalid self-samples (< 0.5%).

This trial was not designed to estimate differences in the proportions of CIN2+ lesions between the intervention groups; thus, observed differences should be interpreted with caution.

### Comparison with other studies

The participation rate in the directly mailed group (38.0%) was higher than in other comparable Dutch trials that achieved participation rates of 26.6 to 30.8% at 12 months [38, 39]. This may be explained by the fact that the Dutch women offered self-sampling were not informed about the possibility of having regular cytology screening at their GP. In the opt-in group, the participation rate (30.9%) was also higher than in previous opt-in trials that report participation rates of 8.7 to 24.5% [19, 20]. This may be due to different definitions of non-participants, differences in the time of participation assessment (range 3 to 12 months), and timely opt-in procedures which made it easier for women to participate. Recently, a Danish opt-in study that targeted women being unscreened for ≥4–6 years achieved a similar participation rate of 30.0%, assessed in a range of 7 to 18 months [40].

Even though we found participation rates in the intervention groups to be slightly lower among the oldest women, the possibility of self-sampling increased participation in all age groups. This suggests that self-sampling is a suitable strategy across different age groups. It is expected that implementation of self-sampling in the Central Denmark Region screening program would increase the overall participation rate among invited women aged 30–64 years by an extra 2% or 5% for the opt-in or direct mail strategy, respectively. The latter figure is similar to the extra 5.2% achieved in a previous Dutch trial that used direct mailing of the self-sampling kit together with a second reminder [38].

Almost one third (32.3%) of the women ordering a self-sampling kit in the opt-in group did not return it. This suggests that intention to be screened was present, but something made the women fail to return the sample. We have no clear explanation for this, but other opt-in studies have reported a similar tendency (range 11% to 39%) [19, 40]. Thus, both mailing self-sampling kits directly and the opt-in procedure result in loss of kits. This should be taken into consideration when choosing the self-sampling invitation strategy and the self-sampling device. Using the webpage and sending a text message were more commonly used ways of ordering the kit than phone and e-mail. This result is in line with the Danish opt-in study, which reported that ordering via a webpage was considered far more acceptable than ordering by phone or e-mail (37% vs 1% vs < 1%, respectively) [40].

Among women receiving the self-sampling offer, we also found that 18.6% of women in the directly mailed group still chose to approach a GP for a cervical cytology sample, while this was the case for even more women (22.6%) in the opt-in group. These findings suggest that self-sampling may be most effective if it is combined with other strategies, i.e. not used as the only option.

The effectiveness of self-sampling depends, among other things, on its capacity to recruit hard-to-reach women at increased risk of developing cervical cancer. Our results show that the direct mailing strategy was superior to the opt-in strategy and the standard second reminder in terms of higher participation among un- or underscreened women, but also among women who had not participated in previous screening rounds. This finding is supported by other trials that used the direct mail invitation strategy [17, 18]; they report that underscreened women were more likely to participate when offered self-sampling (range 10 to 39%) than when sent an invitation/reminder for regular cytology screening (range 4.5 to 9.0%). Thus, self-sampling may have the potential to reduce the earlier documented social inequalities in cervical cancer screening participation [41]. An up-coming registry study based on data from this trial will further explore this issue. These perspectives together with the cost-effectiveness of self-sampling should also be taken into account before planning a general rollout of self-sampling in the routine screening program.

An efficient self-sampling strategy also depends on a high level of compliance with follow-up among HPV-positive self-samplers. Compliance with cervical cytology triage at the GP after a HPV positive self-sample was high in this trial (90.7%); and when the relatively short follow-up measure was extended, the “long-term” follow-up was 95.8% within 180 days. This was higher than was found in previous Dutch trials that recorded follow-up of 89% to 90% at 18 months with a comparable triage protocol [38, 39]. Most importantly, our results show that high compliance with follow-up could be achieved in a real-world screening setting without an intensive follow-up protocol as used in other trials [19, 20]. HrHPV infections were detected in 13.0% of the self-sampling participants. This prevalence was higher than was found in an Australian trial (8.5%) that targeted never- or underscreened women aged 30–69 years using the same HPV test [42]. The hrHPV prevalence among self-samplers (13.0%) was only slightly lower than, but not significantly different from, the 16.2% observed in a HPV screening study that included Danish women aged 30–65 years undergoing regular cytology screening using the Cobas® 4800 HPV test [43].

Although our trial was not scaled to evaluate the effect of the self-sampling initiatives on the detection of CIN2+ lesions, we saw a clear tendency: the proportion of CIN2+ lesions per 1000 invited women was higher in the directly mailed and opt-in group than in the control group*.* This finding might be interpreted as an early indicator for the expected impact on cervical cancer prevention if self-sampling was to be introduced. It should be noted that there seemed to be a difference in CIN2+ detection between the directly mailed and opt-in group, even though four of the five women who were not followed up within 180 days were in the directly mailed group.

The higher detection of CIN2+ among self-sampling participants than among regular cytology participants in the control group, also shown in previous trials [44, 45], may be explained by the fact that primary hrHPV testing is more sensitive than cytology testing for detecting CIN2+ [46]. Assuming that the background risk for CIN2+ is increased among un-or underscreened women [38], the improved coverage of these women when self-sampling was offered could be another explanation for the increased CIN2+ detection found among self-samplers.

## Conclusions

Direct mailing of self-sampling kits to non-participating women was the most effective invitation strategy for increasing participation. Using timely opt-in procedures yielded a limited participation increase compared with a standard second reminder to attend regular cytology screening. Our trial shows that it is feasible to implement HPV self-sampling into the Danish cervical cancer screening program.

Self-sampling has the potential to improve the effectiveness of the program in the Central Denmark Region by increasing the overall participation by an extra 2 to 5% among invited women. Furthermore, it seems that self-sampling motivates hard-to-reach women to re-engage with the screening program. A high compliance with follow-up testing was seen. Offering non-participating women the possibility of HPV self-sampling as an alternative to regular cytology screening should be considered.

## Abbreviations

AGC: Atypical glandular cells
AIS: Adenocarcinoma In Situ
ASC-H: Atypical squamous cells cannot exclude HSIL
ASC-US: Atypical squamous cells of undetermined significance
CIN2: Cervical intraepithelial neoplasia of grade 2 or worse (CIN2+)
CPR: Civil personal registration
DPDB: Danish Pathology Data Bank
GP: General practitioner
HPV: Human papillomavirus
hrHPV: High-risk human papillomavirus
HSIL: High-grade squamous intraepithelial lesion
LSIL: Low-grade squamous intraepithelial lesion
PCR: Polymerase chain reaction
PD: Participation difference
